# Identification and analysis of endoplasmic reticulum stress-related biomarkers in chronic sinusitis with nasal polyps using bioinformatics approaches

**DOI:** 10.1097/MD.0000000000049336

**Published:** 2026-06-12

**Authors:** Yue Zhai, Hanli Lu, Qiu Ding

**Affiliations:** a Department of Otorhinolaryngology, The Affiliated Chuzhou Hospital of Anhui Medical University, Chuzhou, Anhui, China; b Department of Orthopaedics, The Affiliated Chuzhou Hospital of Anhui Medical University, Chuzhou, Anhui, China.

**Keywords:** chronic rhinosinusitis with nasal polyps, oxidative stress, machine learning, weighted gene co-expression network analysis, bioinformatics

## Abstract

Chronic rhinosinusitis with nasal polyps (CRSwNP) is a prevalent inflammatory disease of the sinuses that significantly diminishes patients’ quality of life. Endoplasmic reticulum stress (ERS) is strongly associated with the initiation and progression of numerous inflammatory diseases. This study aimed to identify ERS-related hub genes in CRSwNP using bioinformatics approaches. We combined the GSE136825 and GSE179265 datasets. Subsequently, we performed differential expression analysis and weighted gene co-expression network analysis. The genes identified were then cross-referenced with those associated with ERS, allowing us to identify ERS-related differentially expressed genes in CRSwNP. We employed 4 machine learning algorithms: support vector machine, random forest, extreme gradient boosting, and least absolute shrinkage and selection operator. These methods facilitated the identification of hub genes, which were then used to construct a nomogram for disease prediction. Twenty-eight genes related to ERS showed significant expression differences (*P* < .05). By overlapping the genes selected from 4 machine learning algorithms, we identified 3 hub ERS-differentially expressed genes: ATP2A3, ACTC1, and DES. The nomogram prediction model built on these pivotal genes demonstrated strong predictive performance. This study offers new insights into the molecular mechanisms of CRSwNP and suggests potential new avenues for early diagnosis and targeted treatment in future clinical practice.

## 1. Introduction

Chronic rhinosinusitis (CRS) is defined as a chronic inflammatory disease of the sinus mucosa lasting more than 12 weeks,^[1]^ with an overall prevalence of 8% in the Chinese population.^[2]^ The etiology of CRS is complex, and it is typically classified clinically into 2 categories: CRS with nasal polyps (CRSwNP) and CRS without nasal polyps.^[3]^ Increasing evidence indicates that CRSwNP and CRS without nasal polyps differ significantly in terms of pathogenesis, treatment, and prognosis. Patients with CRSwNP are more likely to have comorbid conditions such as allergic rhinitis and asthma.^[4]^ Additionally, there is a higher infiltration of eosinophils and type 2 inflammatory cytokines in the tissues of CRSwNP patients, making the condition more difficult to manage with a higher recurrence rate.^[5]^ In cases of eosinophilic CRSwNP, the recurrence rate can exceed 50%.^[6]^

It is currently believed that the pathogenesis of CRSwNP is associated with various factors, including anatomical abnormalities of the ostiomeatal complex, allergies, infections from bacteria, viruses, and fungi, bacterial superantigens, aspirin intolerance, and ciliary dyskinesia. However, the exact mechanisms remain unclear. A hallmark of CRSwNP is prolonged inflammation of the nasal and paranasal sinuses mucosa. Patients typically present with symptoms such as nasal congestion, purulent discharge, and hyposmia, significantly impacting their quality of life and work efficiency.^[7]^ These symptoms can lead to increased levels of anxiety and depression.^[8,9]^ The persistent nature of the illness, combined with repeated treatments (both medical and surgical) places substantial physical, psychological, and financial burdens on patients.^[7,10]^ Current treatment options for CRSwNP primarily include surgical interventions, local or systemic corticosteroids, anti-infective and anti-allergic therapies, mucolytics, and targeted biologic therapies.^[1]^ However, the recurrence rate of CRSwNP remains high. There is an urgent need to deeply understand the molecular and genetic mechanisms of CRSwNP to provide further clinical diagnosis and treatment.

The endoplasmic reticulum (ER) is one of the important organelles in cells, playing a crucial role in the synthesis, modification, and transport of various substances such as proteins and lipids. Under stress conditions like hypoxia, nutrient deprivation, and lactic acidosis, the normal function of the ER is disrupted, leading to the accumulation of misfolded and unfolded proteins within the ER. This accumulation ultimately triggers ER stress (ERS).^[11,12]^ Research has shown a close link between ERS and inflammation.^[13]^ For instance, studies indicate that *Staphylococcus aureus* enterotoxin B can increase the production of reactive oxygen species in human nasal epithelial cell lines, thereby activating ERS and playing a significant role in CRSwNP.^[14]^ Additionally, ERS can exacerbate inflammation in CRSwNP through the transcription factor XBP1.^[15]^ However, the functions of ERS-related genes in CRSwNP remain unclear. In this study, we aim to analyze the role of ERS-related genes in CRSwNP and explore reliable biomarkers, which will provide new insights for both research and clinical treatment of CRSwNP.

In recent years, advances in high-throughput genome, transcriptome sequencing, and even spatial transcriptome sequencing technologies at the single-cell level have promoted rapid progress in the fields of biology, immunology, and oncology. Machine learning algorithms have promoted the transition from gene discovery to practical diagnostic applications, can effectively identify key biomarkers in genetic data, capture complex gene-disease associations through algorithmic modeling, and ultimately build high-performance diagnostic models with clinical practicability.^[16]^

In this study, we hypothesized that ERS-related differentially expressed genes (DEGs) are involved in the pathogenesis of CRSwNP and may serve as potential biomarkers. To test this hypothesis, we used data in the gene expression omnibus (GEO) database to conduct multiple bioinformatics analyses, including machine learning, to identify key genes related to ERS and build a prediction model for CRSwNP.

## 2. Materials and methods

### 2.1. Data download and processing

We downloaded the datasets GSE136825,^[17]^ GSE179265,^[18]^ GSE36830,^[19]^ and GSE72713^[20]^ from the GEO database (https://www.ncbi.nlm.nih.gov/geo/). The GSE136825 dataset includes nasal polyp tissues from 42 CRSwNP patients and inferior turbinate tissues from 28 healthy controls. The GSE179265 dataset consists of nasal polyp tissues from 17 CRSwNP patients and uncinate tissues from 7 healthy controls. The GSE36830 dataset comprises nasal polyp tissues from 6 CRSwNP patients and uncinate tissues from 6 healthy controls. Finally, the GSE72713 dataset includes nasal polyp tissues from 6 CRSwNP patients and tissues from 3 healthy controls. We normalized the GSE136825 and GSE179265 datasets, as well as the GSE36830 and GSE72713 datasets, using the “NormalizeBetweenArrays” function from the “limma” package, followed by merging them accordingly. Subsequently, we applied the “ComBat” function from the “sva” package to eliminate batch effects between the 2 datasets. Although the control samples in the datasets are from the inferior turbinate or uncinate process, the mucosa of the inferior turbinate and uncinate process is anatomically continuous, and the types of mucosa are pseudostratified ciliated columnar epithelium, so the difference in gene tissue-specific expression will not affect the subsequent analysis after merging the datasets. This view is further supported by the work of Zhu et al.^[21]^ The merged datasets GSE136825 and GSE179265 were considered the primary dataset, while the combined datasets GSE36830 and GSE72713 were used to validate the analysis results.

Genes related to ERS were obtained from the literature, in which the authors retrieved ERS-associated genes from the GeneCards database and selected genes with a Relevance score > 5 (totaling 1350 genes) for subsequent analysis.^[22,23]^ Using the “venn” and “ggvenn” R packages, the intersection was taken between these 1350 ERS-related genes and the DEGs from the CRSwNP dataset, as well as the core module genes identified by weighted gene co-expression network analysis (WGCNA) analysis, to obtain ERS-DEGs associated with CRSwNP. Ethics approval or consent was not required as the data used in this study were obtained from publicly available databases.

### 2.2. Difference analysis

We used the R package “limma” to identify DEGs between the CRSwNP group and the control group, applying thresholds of adjusted *P* values (Benjamini–Hochberg method) < .05 and log2FC ≥ 1 or ≤ −1. The R packages “ggplot2” and “pheatmap” were utilized to create volcano plots and heatmaps for the identified DEGs.

### 2.3. WGCNA

We used the R package “WGCNA” to construct modules of co-expressed genes that are highly correlated. First, we selected an appropriate power value to create a gene adjacency matrix, which was then converted into a topological overlap matrix (TOM) with a soft threshold of 3. The dissimilarity TOM (1-TOM) was used as input for gene clustering analysis. Next, we constructed a hierarchical clustering dendrogram to categorize genes with similar expressions into various modules (minModuleSize = 100, mergeCutHeight = 0.5). Finally, we calculated the correlation between feature genes in each module and CRSwNP, selecting the module with the highest correlation as the hub module for this study.

### 2.4. Machine learning models

Based on the expression profiles of 28 ERS-DEGs, we constructed 3 machine learning models: extreme gradient boosting (XGB), random forest (RF),^[24]^ and support vector machine (SVM)^[25]^ using the R package “caret.” These models were employed to perform “feature importance evaluation and ranking” for each gene based on their respective built-in mechanisms. Within each model, an importance score was calculated for each gene, aiming to identify the most critical genes from different algorithmic perspectives. A total of 94 samples were randomly divided into a validation group (30%, n = 27) and a training group (70%, n = 67). The “DALEX” package was utilized to interpret the aforementioned machine learning models. The “caret” R package was used to define the parameters for these models, which were evaluated through 10-fold cross-validation. We then employed the “pROC” package to illustrate the area under the Receiver Operating Characteristic (ROC) curve. Finally, we presented the 10 most significant genes identified by each of the 3 models.

Meanwhile, for the expression profiles of the 28 ERS-DEGs, we performed least absolute shrinkage and selection operator (LASSO) regression analysis using the “glmnet” package^[26]^ to identify DEGs associated with ERS. The core objective of LASSO regression analysis is feature selection, which automatically screens for the most relevant and strongest-signaling genes by shrinking their coefficients.

To obtain more reliable results, we intersected the genes selected by the LASSO algorithm with the top 10 most significant genes from each of the 3 machine learning models (XGB, RF, and SVM). The 3 overlapping genes were identified as hub ERS-DEGs. This approach aimed to reduce bias from any single model through cross-validation by multiple algorithms, thereby screening for high-confidence hub ERS-DEGs.

We conducted a Spearman correlation analysis on the 3 hub ERS-DEGs to assess their interrelationships. Additionally, we utilized the “RCircos” package to create a map of 23 chromosomes, illustrating the locations of these 3 genes on the chromosomes.

### 2.5. Analysis of immune infiltration

CIBERSORT analyzes the cellular composition of tissues based on an input reference gene set.^[27]^ The LM22 gene feature matrix, which includes 547 genes, can distinguish between 22 types of human immune cells and was used as the annotation gene set. We employed the CIBERSORT algorithm along with the LM22 feature matrix to calculate the relative infiltration proportions of immune cells in both the CRSwNP and control groups, visualizing the results using the R package “ggplot2.” CIBERSORT employs Monte Carlo sampling to estimate the *P* value, which evaluates the confidence level of the deconvolution results for each sample. In this study, only samples with *P* values < .05 were included in the final analysis to ensure the robustness and accuracy of the inferred immune cell proportions. The correlations between the 3 hub genes and immune cells were analyzed using the “psych” package, and the results were visualized with “ggplot2.”

### 2.6. Gene set variation analysis (GSVA)

We conducted GSVA functional enrichment analysis using the R packages “GSVA” and “GSEABase” to investigate differences in biological process activity and pathways between the CRSwNP and control groups.^[28]^ The “limma” package was used to identify differential biological functions and expression pathways by comparing GSVA scores between the 2 groups. A GSVA score t-value > 2 was considered statistically significant.

### 2.7. Enrichment analysis

Gene ontology (GO)^[29]^ functional enrichment and Kyoto Encyclopedia of Genes and Genomes (KEGG)^[30]^ pathway analysis were conducted to identify the biological functions and pathways associated with 28 DEGs related to ERS. GO annotations are categorized into 3 types: biological process, cellular component, and molecular function. We performed GO and KEGG analyses using the R packages “clusterProfiler,” “org.Hs.eg.db,” and “enrichplot.” considering pathways with a *P* value of < .05 as significant.

### 2.8. Construction of a prediction model

Based on the 3 hub ERS-DEGs, we constructed a nomogram model using the “rms” R package to evaluate the incidence of CRSwNP. We then assessed the predictive performance of this model through decision curve analysis (DCA), calibration curves, and ROC curves. The nomogram is a predictive model based on multivariate logistic regression analysis, where each gene is assigned a score according to the magnitude of its regression coefficient. Specifically, a higher score indicates a greater contribution of that gene to the final risk assessment for disease prediction. The variance inflation factor was applied to diagnose multicollinearity, ensuring that no severe multicollinearity exists among the variables. Following this, we validated the model through ROC analysis using the combined expression profiles from GSE36830 and GSE72713.

### 2.9. Statistical analysis

We conducted statistical analyses using the aforementioned software packages and R version 4.3.3 (R Foundation). Continuous variables are presented as mean ± standard deviation, and Spearman correlation analysis was used to determine relationships between variables. A *P* value of < .05 was considered statistically significant. Specifically, * indicates *P* < .05, ** indicates *P* < .01, and *** indicates *P* < .001.

## 3. Results

### 3.1. Data processing and differential analysis

**Figure 1.**
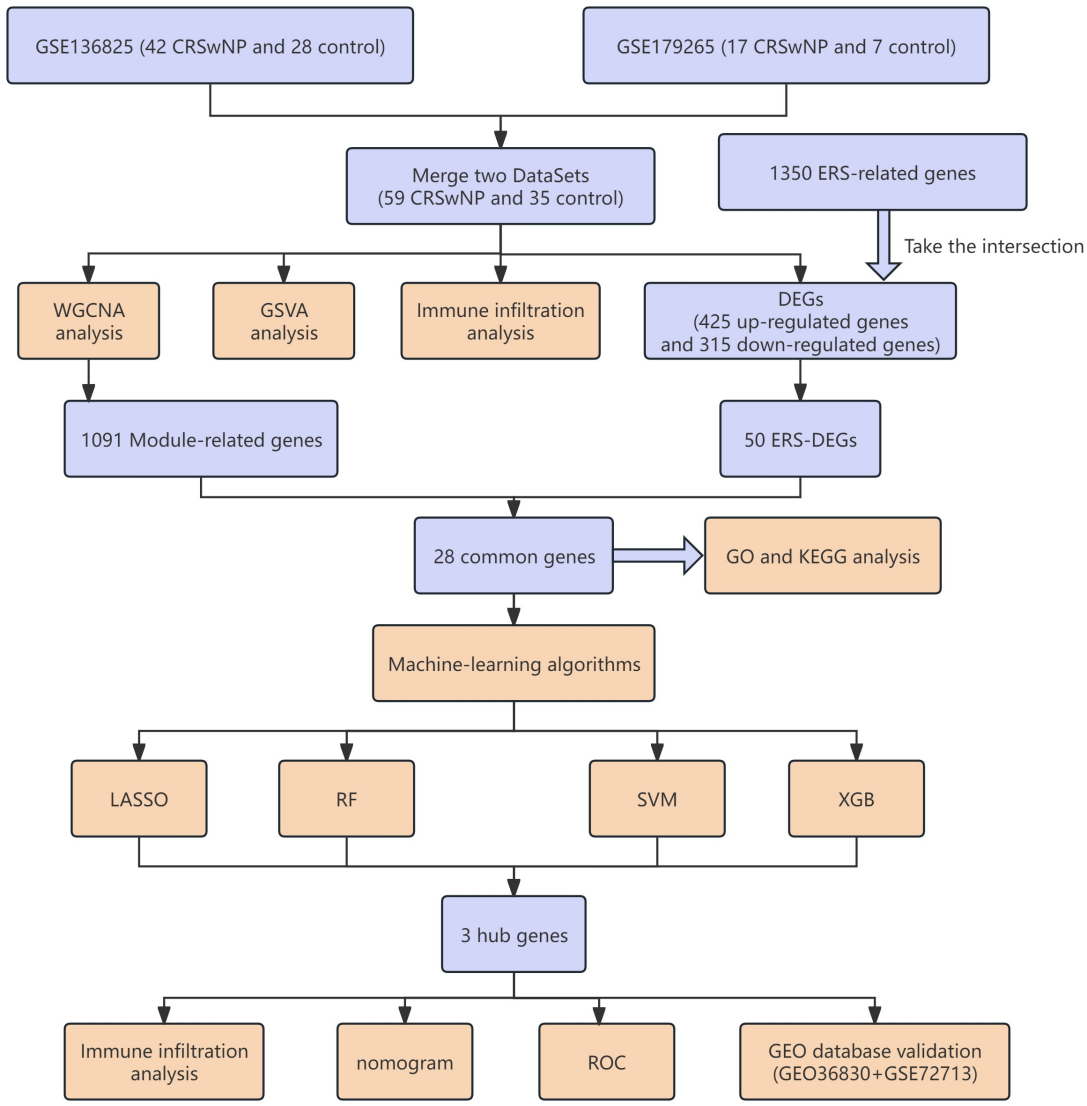
Technology roadmap. CRSwNP = chronic rhinosinusitis with nasal polyps, DEGs = differentially expressed genes, ERS = endoplasmic reticulum stress, GEO = gene expression omnibus, GO = gene ontology, GSVA = gene set variation analysis, KEGG = Kyoto Encyclopedia of Genes and Genomes, LASSO = least absolute shrinkage and selection operator, RF = random forest, ROC = receiver operating characteristic, SVM = support vector machine, WGCNA = weighted gene co-expression network analysis, XGB = extreme gradient boosting.

**Figure 2.**
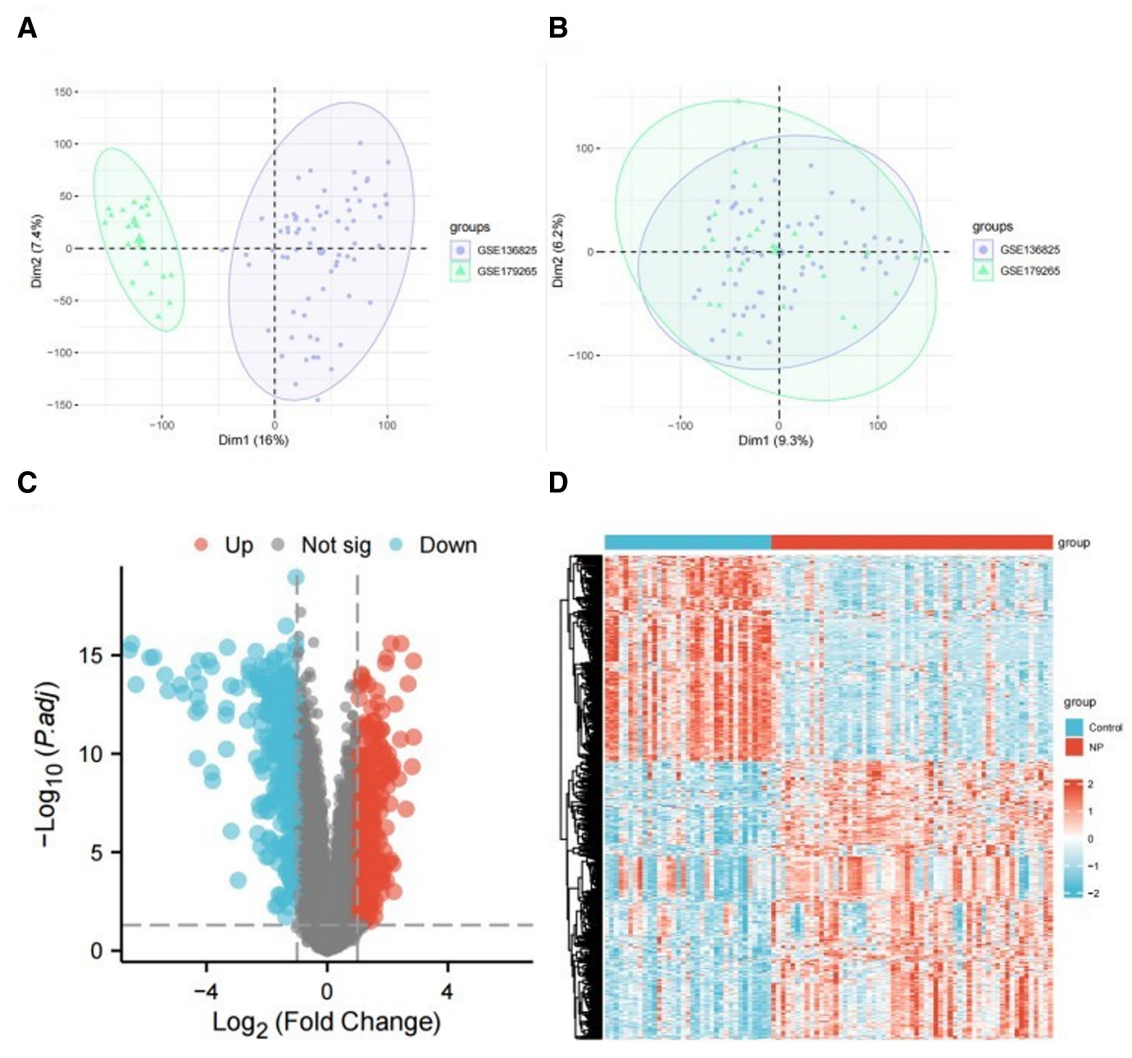
Identification of differentially expressed genes. (A) PCA plot of expression profiles before merging datasets GSE136825 and GSE179265. (B) PCA plot of expression profiles after merging datasets GSE136825 and GSE179265. (C) A volcano plot of DEGs after merging datasets. (D) Heatmap of DEGs after merging datasets.. DEG = differentially expressed genes, PCA = principal component analysis.

The technical roadmap for this study is illustrated in Figure 1. We obtained the GSE136825 and GSE179265 datasets from the GEO database, which included a total of 59 CRSwNP samples and 28 normal control samples. After merging the 2 datasets, we normalized the data and removed batch effects, followed by visualization using principal component analysis (Figs. 2A and 2B). Subsequently, we performed a differential analysis on the merged dataset, applying thresholds of adjusted *P* values (Benjamini–Hochberg method) < .05 and log2FC ≥ 1 or ≤ −1 to identify DEGs. This analysis revealed 425 upregulated genes and 315 downregulated genes, which were visualized using volcano plots and heatmaps (Figs. 2C and 2D).

### 3.2. Characteristics of immune infiltration and functional pathways in CRSwNP

**Figure 3.**
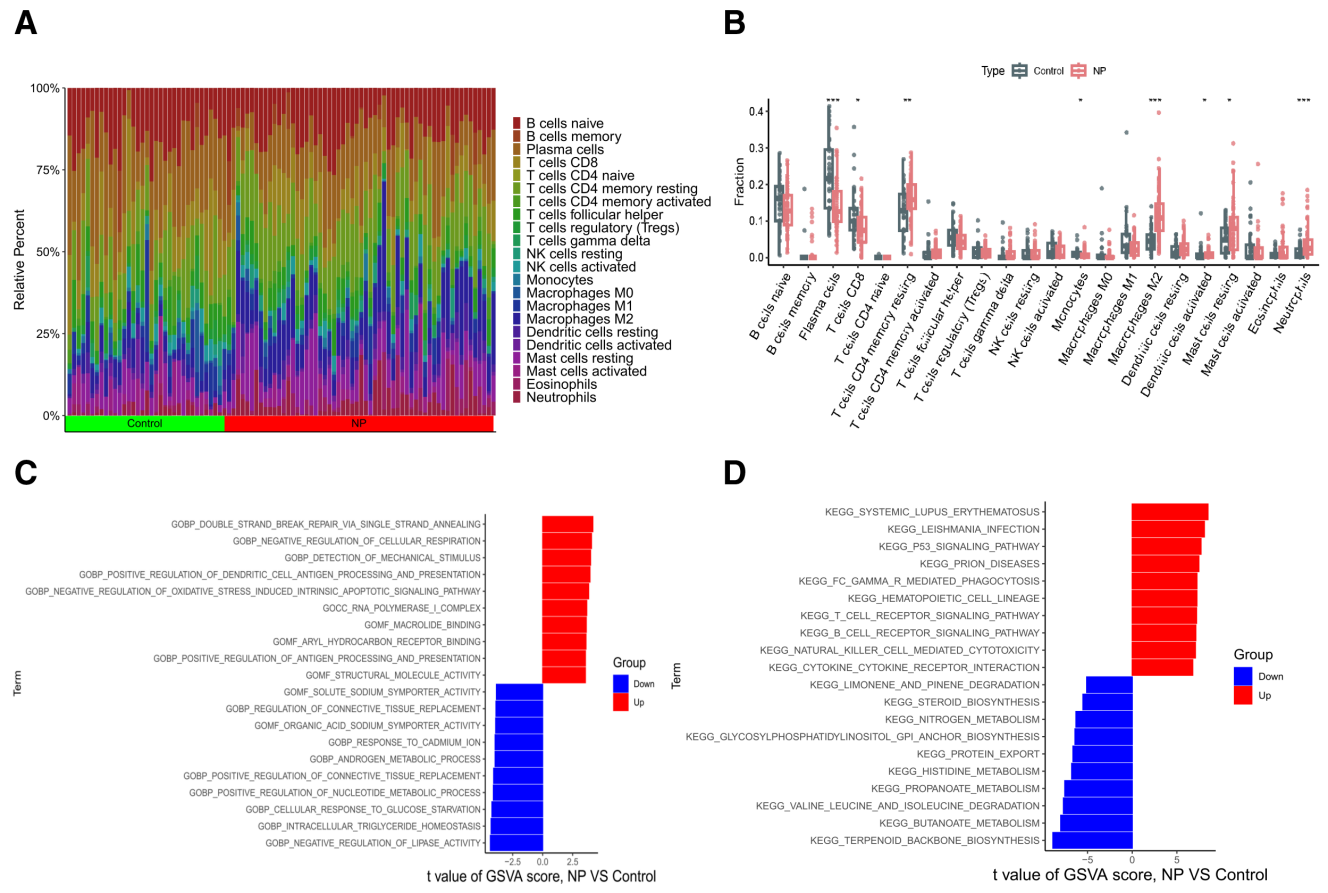
Immune characteristics and biological processes of CRSwNP and normal groups. (A) and (B) The relative abundance and boxplot of 22 infiltrating immune cells between the 2 phenotypes. (C) and (D) Uncovered the KEGG and GO enrichment pathways in the 2 phenotypes. CRSwNP = chronic rhinosinusitis with nasal polyps, GO = gene ontology, GSVA = gene set variation analysis, KEGG = Kyoto Encyclopedia of Genes and Genomes, NP = nasal polyps, VS = versus.

Based on the CIBERSORT algorithm, we performed immune infiltration analysis to calculate the relative infiltration abundance of 22 types of immune cells between CRSwNP samples and normal controls. Compared to the control group, CRSwNP tissues exhibited higher infiltration levels of resting memory CD4+ T-cells, M2 macrophages, activated dendritic cells, resting mast cells, and neutrophils. Conversely, plasma cells, CD8 T-cells, and monocytes had lower proportions in CRSwNP samples (Figs. 3A and 3B). This suggests that immune system activation may be linked to the pathogenesis of CRSwNP.

Besides, we conducted a GSVA analysis to examine the functional differences between the CRSwNP and normal control groups. Our findings indicated that CRSwNP is primarily associated with pathways related to cellular respiration, oxidative stress-induced apoptosis, the p53 signaling pathway, systemic lupus erythematosus, and T-cell and B-cell receptor signaling. In contrast, pathways involving protein export, valine degradation, nucleotide metabolism regulation, and intracellular triglyceride homeostasis were downregulated in CRSwNP samples (Figs. 3C and 3D).

### 3.3. WGCNA

**Figure 4.**
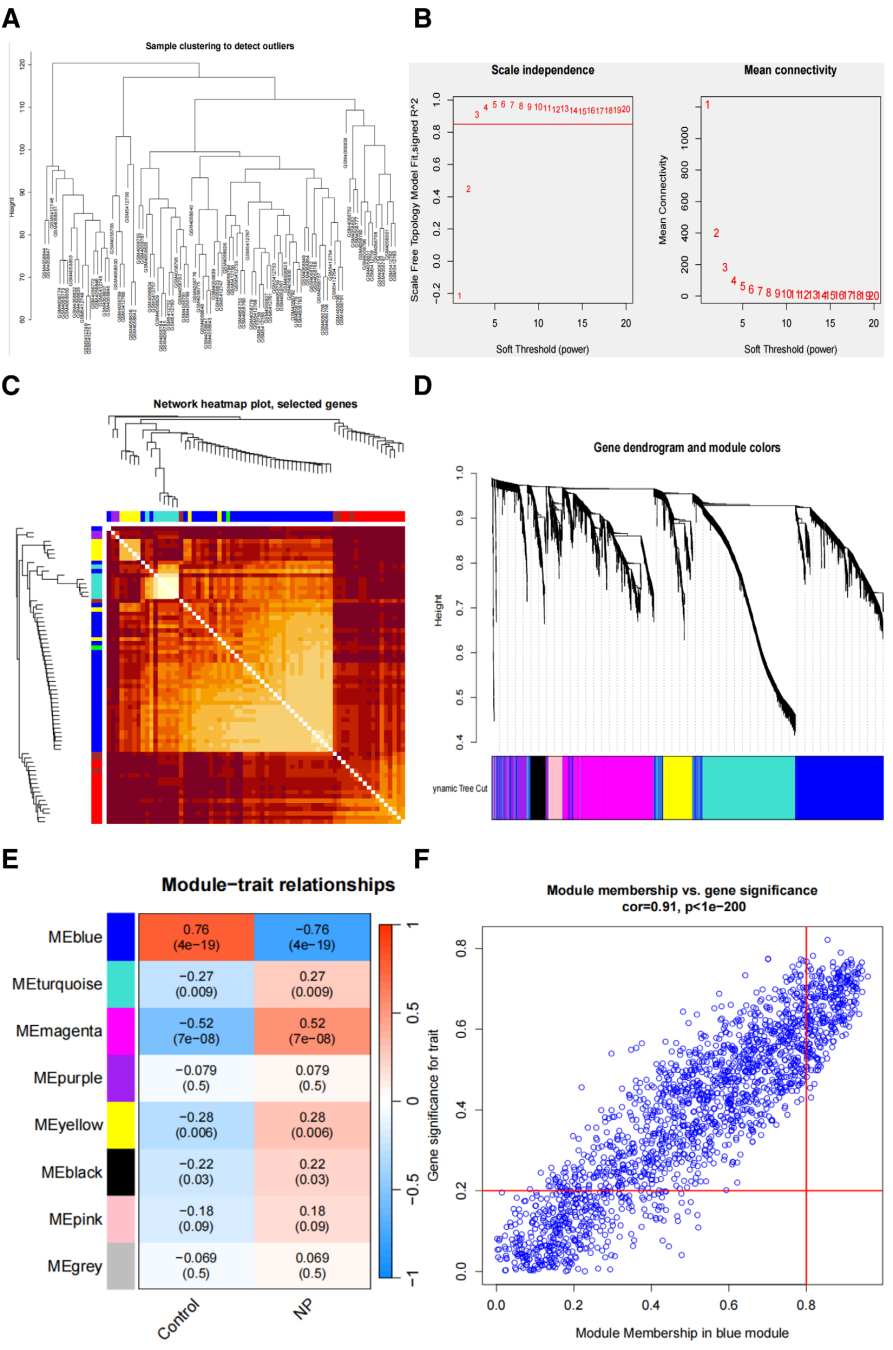
Construction of the co-expression network for differentially expressed genes in CRSwNP. (A) Sample clustering to detect outliers; no obvious outliers in the figure. (B) Network topology analysis for the soft thresholding powers. (C) Heatmap plots of the correlations between modules. (D) Dendrogram of clustered genes with topologically overlapping-based variability and specified module colors. (E) Module trait correlations. Each column matches a trait, and each row matches a module eigengene. Each unit cell includes the corresponding *P* value and correlation. It can be seen that the blue module has the highest correlation. (F) A scatterplot of MM in the blue module vs GS for CRSwNP. CRSwNP = chronic rhinosinusitis with nasal polyps, GS = gene significanc, MM = module membership.

WGCNA analysis can identify gene sets highly correlated with phenotypic traits through algorithms distinct from the aforementioned differential expression analysis. To further identify disease-related genes in the CRSwNP and control groups, we conducted a WGCNA analysis. Among all genes, we selected the top one-sixth of genes with the highest variability for further analysis. Using the “flashClust” package, we performed a clustering analysis on the samples, as shown in Figure 4A. Based on the scale-free topology criterion (*R*^2^ > 0.85), we set the optimal soft threshold to 3 to construct a scale-free network (Fig. 4B). Next, we employed a dynamic cutting algorithm to identify 8 distinct gene modules associated with CRSwNP and created a 1-TOM heatmap (Figs. 4C and 4D). We then calculated the correlation between module characteristic genes within each gene module and clinical traits (Fig. 4E). The blue module exhibited the strongest correlation with CRSwNP (*r* = 0.76, *P* = 4.0e−19) and was therefore selected for further analysis, containing 1091 genes. Finally, we plotted a scatter diagram of module membership in the blue module against gene significance for CRSwNP, revealing a significant positive correlation between the two (Fig. 4F).

### 3.4. Selection of differential genes associated with ERS (ERS-DEGs)

**Figure 5.**
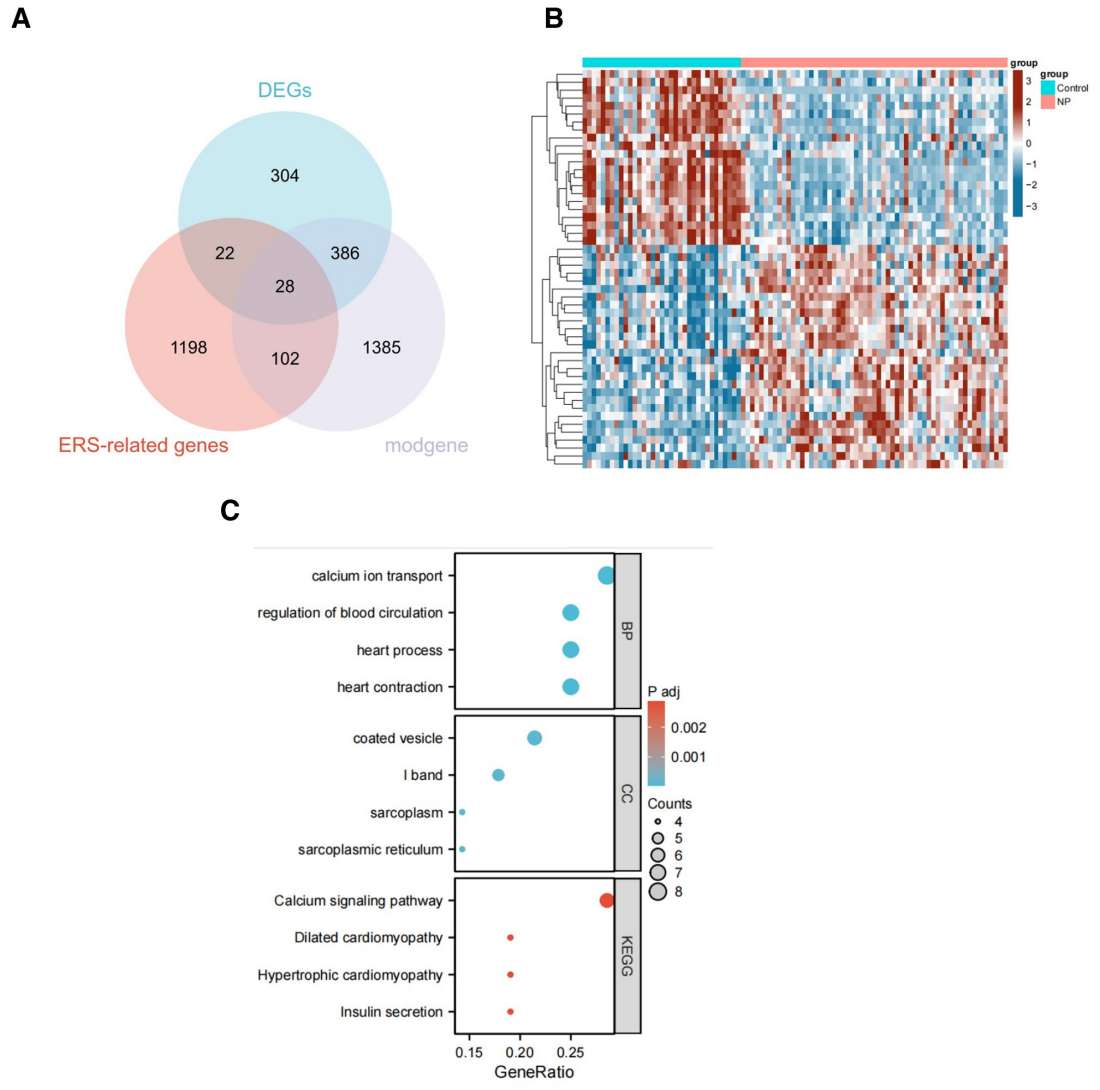
Selection of ERS-DEGs. (A) Venn diagram showing the intersection of DEGs with the 1091 genes from the blue module and the 1350 ERS-related genes, resulting in 28 ERS-DEGs. (B) Heatmap of 28 ERS-DEGs. (C) Bubble plot showing the GO and KEGG enrichment analysis of the 28 ERS-DEGs. BP = biological process, CC = cellular component, DEGs = differentially expressed genes, ERS = endoplasmic reticulum stress, GO = gene ontology, KEGG = Kyoto Encyclopedia of Genes and Genomes.

To understand the expression of ERS-related genes among the DEGs associated with CRSwNP, we intersected the DEGs obtained from the above differential analysis with the 1091 genes from the blue module in WGCNA and 1350 ERS-related genes. The resulting 28 ERS-DEGs are not only highly associated with CRSwNP but also strongly correlated with ERS (Fig. 5A). Their expression profile heatmap was generated using the R package “pheatmap” (Fig. 5B).

We performed GO and KEGG enrichment analyses on these 28 ERS-DEGs using the “clusterProfiler” package. Entries with a *P* value < .05 were considered statistically significant. The GO analysis indicated that these 28 genes are primarily involved in regulating processes related to blood circulation and cellular components such as sarcoplasmic membranes. The KEGG analysis revealed that these genes are mainly enriched in the calcium signaling pathway, insulin secretion, and other signaling pathways (Fig. 5C).

### 3.5. Identification of hub ERS-DEGs

**Figure 6.**
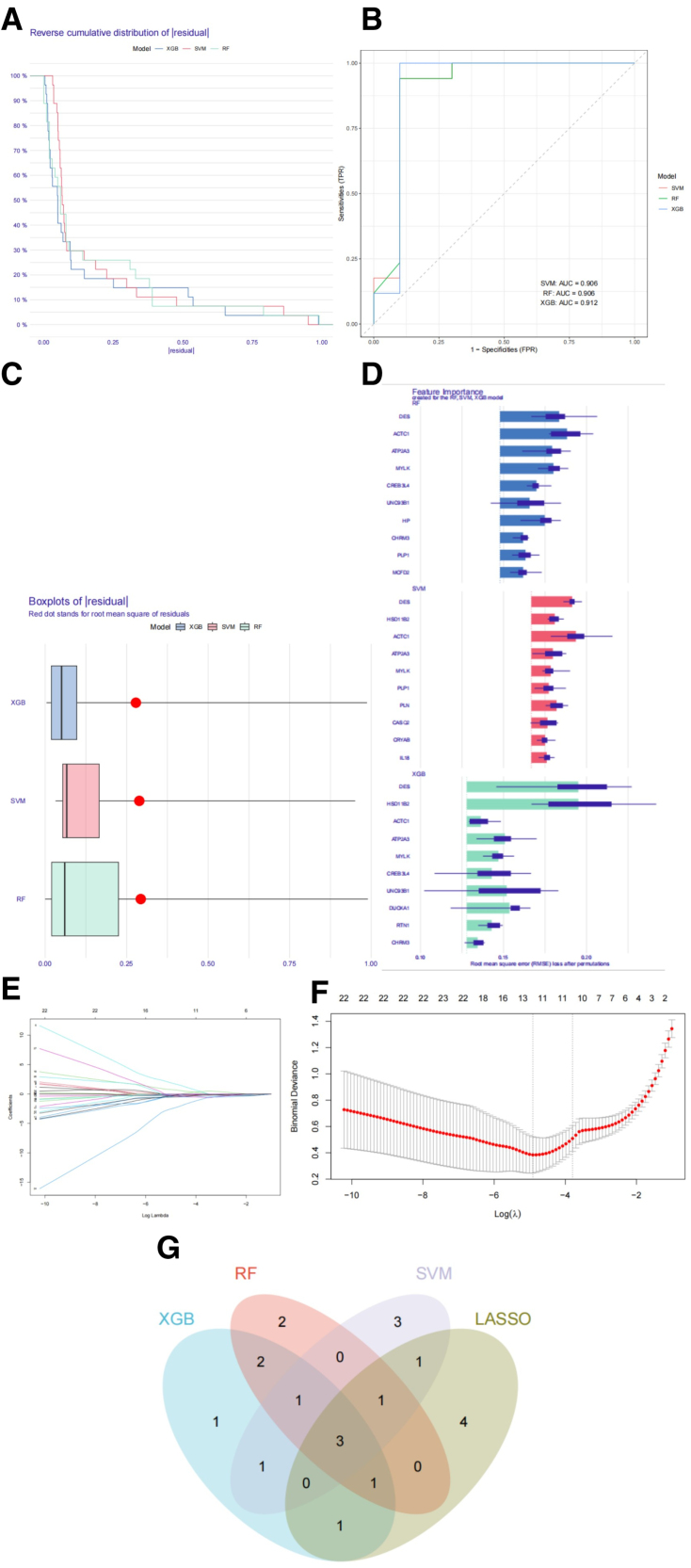
Identification of hub ERS-DEGs. (A) Reverse cumulative distributions of the RF, SVM, and XGB models. (B) ROC curves analysis of the RF, SVM, and XGB models. (C) The residuals of 3 models were shown in the boxplot. (D) Top 10 important genes in the RF, SVM, and XGB models. (E) In the LASSO analysis, 10-fold cross-validation was performed within the 28 ERS-DEGs to select the optimal tuning parameter determined by log(lambda). (F) Regression coefficients of genes obtained from LASSO regression analysis and results from stepwise cox regression analysis. (G) Venn diagram showing 3 hub ERS-DEGs in the intersection between RF, SVM, XGB, and LASSO models. AUC = area under the curve, DEGs = differentially expressed genes, ERS = endoplasmic reticulum stress, FPR = false positive rate, LASSO = least absolute shrinkage and selection operator, RF = random forest, ROC = receiver operating characteristic, SVM = support vector machine, TPR = true positive rate, XGB = extreme gradient boosting.

To further identify genes with high predictive value, we constructed models based on the 28 ERS-DEGs identified in the previous step using machine learning algorithms (SVM, RF, and XGB). All 3 models exhibited a low root mean square of residuals (Figs. 6A and 6C). The ROC analysis indicated that the area under the curve for the SVM, RF, and XGB models was 0.906, 0.906, and 0.912, respectively (Fig. 6B). From these machine learning models, we identified the top 10 most important genes for each model (Fig. 6D).

Subsequently, we conducted a LASSO analysis on 28 genes (Fig. 6E and 6F), which identified 11 significant genes: ACTC1, ATP2A3, COL7A1, CREB3L4, CRYAB, DES, DUOXA1, EDN1, EGF, F5, and PLP1. Finally, we intersected the genes identified by 4 methods: SVM, RF, XGB, and LASSO, resulting in the identification of 3 final hub genes: ATP2A3, ACTC1, and DES, as illustrated in Figure 6G. These hub genes will be used to develop subsequent clinical prediction models related to ERS in CRSwNP.

### 3.6. Immune infiltration and correlation analysis of hub ERS-DEGs

**Figure 7.**
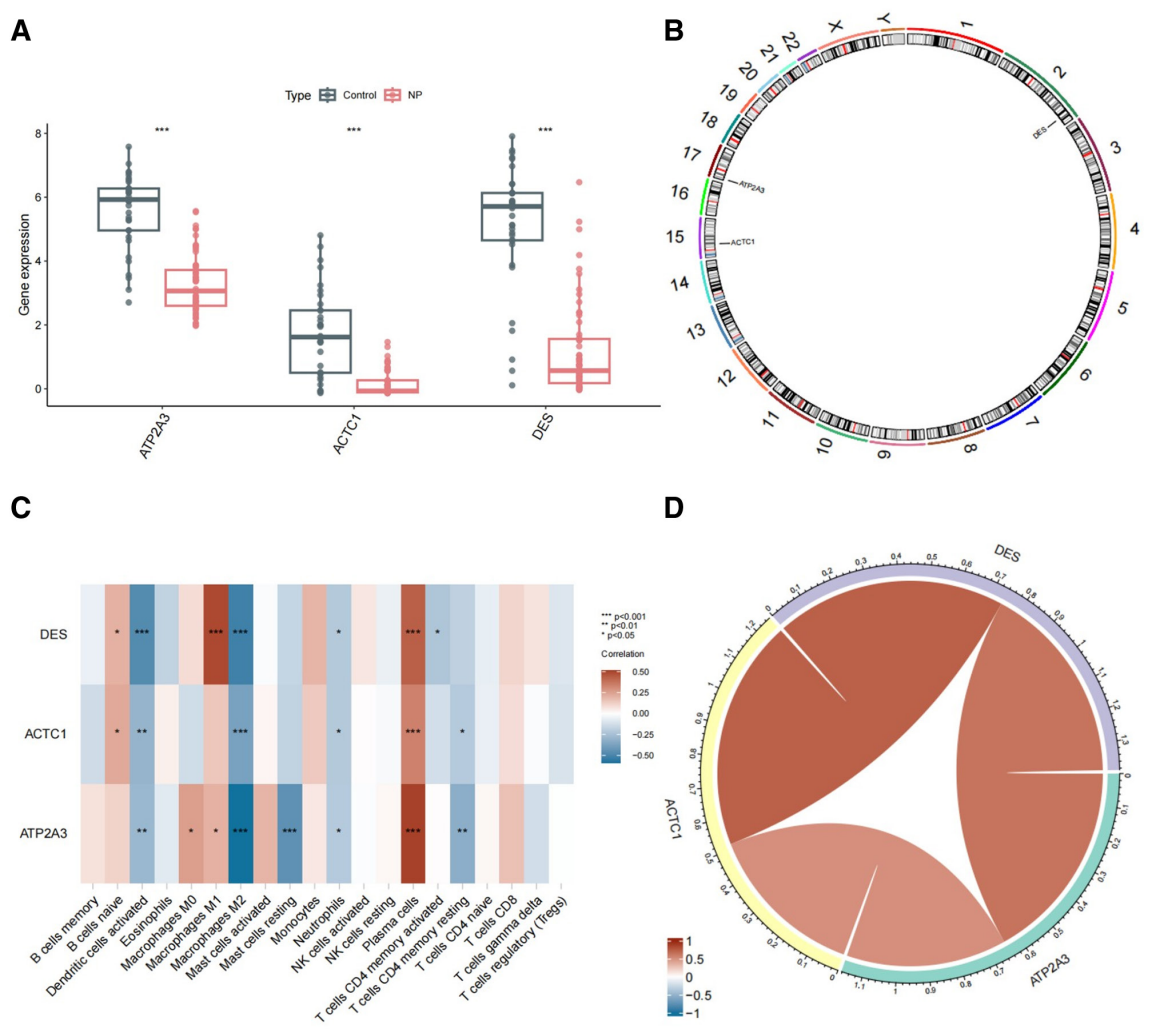
Immune infiltration and correlation analysis of 3 hub ERS-DEGs. (A) Boxplots visualize the expression differences of the 3 hub ERS-DEGs. (B) Location of the 3 hub ERS-DEGs on the chromosome. (C) The relationship between 3 hub ERS-DEGs and immune-infiltrating cells. (D) Gene relationship circle diagram for the 3 hub ERS-DEGs. The red and blue lines represent positive and negative correlations, respectively. Significance levels were indicated by *P < .05, **P < .01, ***P < .001. DEGs = differentially expressed genes, ERS = endoplasmic reticulum stress.

Using 4 methods— (SVM, RF, XGB, and LASSO) we identified 3 hub genes: ATP2A3, ACTC1, and DES. These 3 genes all showed low expression levels in patients with CRSwNP (Fig. 7A). The chromosomal locations of these hub genes are depicted in Figure 7B. Next, we analyzed the correlation between the hub genes and immune-infiltrating cells to investigate their potential role in immune regulation within CRSwNP. The results showed that the 3 hub genes were negatively correlated with macrophages M2, activated dendritic cells, resting mast cells, and Neutrophils, while exhibiting a positive correlation with plasma cells (Fig. 7C). This finding is consistent with the results of the aforementioned immune infiltration analysis, indicating that these genes may play an immunomodulatory role in CRSwNP. Additionally, we conducted correlation analysis among the 3 hub genes to explore their interactions. The results showed that there was a correlation among the 3 hub genes, but the pairwise correlation was <0.8, which reduced the possibility of multicollinearity in the subsequent nomogram model (Fig. 7D).

### 3.7. Development and validation of the predictive model

**Figure 8.**
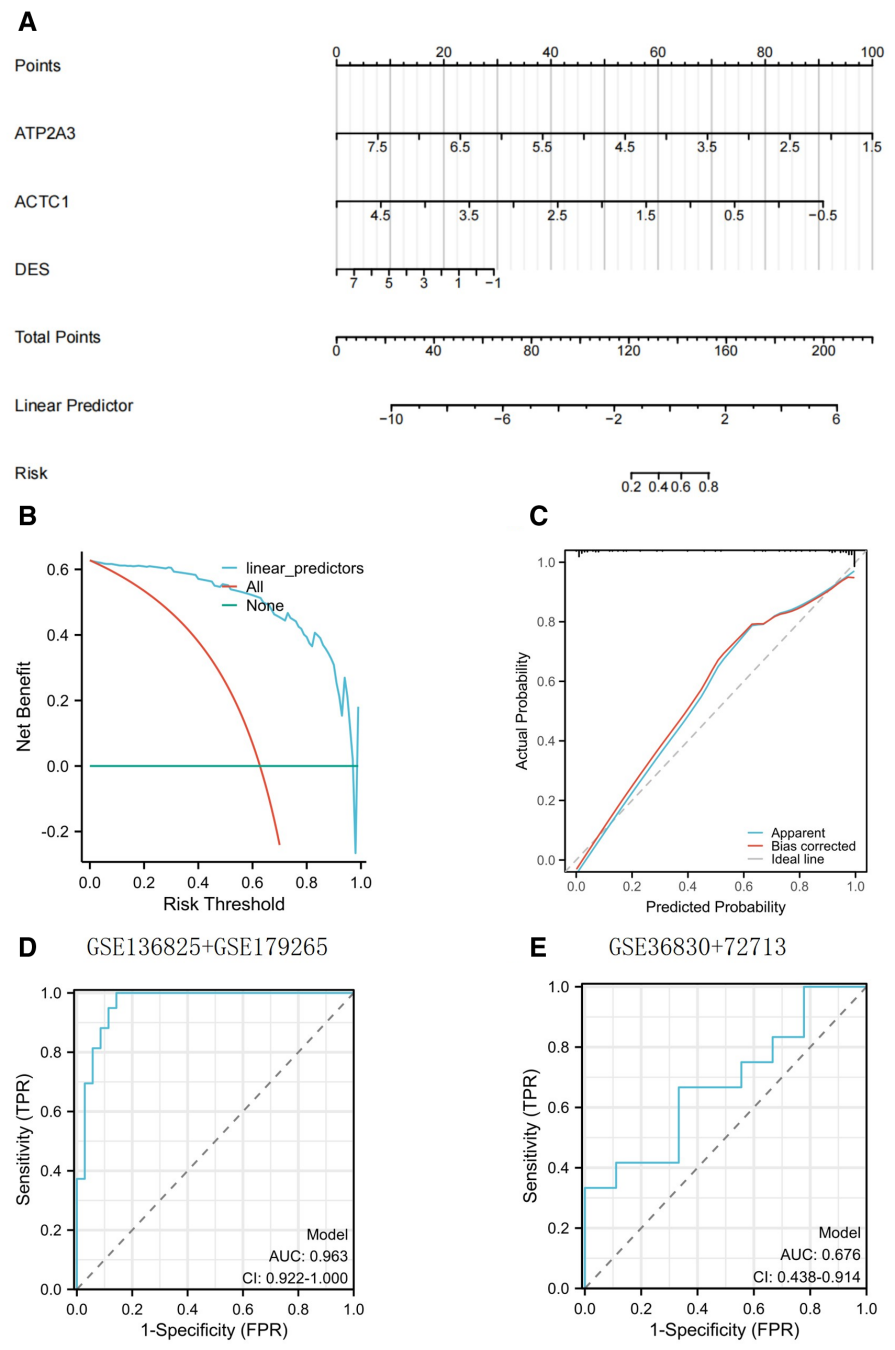
Construction and validation of a prediction nomogram. (A) The nomogram to predict the risk of CRSwNP using the 3 hub ERS-DEGs. Establishment of the (B) DCA and (C) calibration to evaluate the prediction efficacy of the nomogram. ROC curves for the prediction model in the (D) GSE136825 + GSE179265 dataset and (E) GSE36830 + GSE72713 dataset confirm the strong diagnostic performance and robustness of the model. AUC = area under the curve, CI = confidence interval, CRSwNP = chronic rhinosinusitis with nasal polyps, DCA = decision curve analysis, DEGs = differentially expressed genes, ERS = endoplasmic reticulum stress, ROC = receiver operating characteristic.

We selected 3 hub genes (ATP2A3, ACTC1, DES) screened by machine learning to construct the nomogram model (Fig. 8A), which allows us to assess the risk of CRSwNP. Before constructing the model, the pairwise correlation coefficients of all 3 hub genes were below 0.8 (Fig. 7D), and the variance inflation factors were below 4, indicating that there was no serious multicollinearity problem. Subsequent calibration curves and DCA were used to evaluate the predictive performance of the model (Figs. 8B and 8C). The ROC analysis indicated high accuracy for the model (Fig. 8D, area under the curve = 0.963). Additionally, we utilized external datasets GSE36830 and GSE72713 to validate the predictive capability of the model. To minimize the risk of false positives due to small sample sizes in individual datasets, we combined GSE36830 and GSE72713. In the merged dataset, the area under the ROC curve was 0.676, demonstrating good predictive ability (Fig. 8E). Because the sample size of the current CRSwNP data set is not large enough, the prediction performance of the model needs to be further verified by new datasets and patient samples.

## 4. Discussion

CRSwNP is a disease characterized by persistent inflammation of the nasal cavity and sinus mucosa accompanied by polyp growth. It brings a great burden to patients’ health and social economy,^[7,10]^ which has become an increasingly serious public health problem, but its mechanism has not been fully clarified.

Previous studies have indicated that ERS plays a significant role in nasal inflammation such as CRSwNP and allergic rhinitis, which can exacerbate inflammatory responses.^[14,15,31]^ However, our understanding of the mechanisms underlying ERS in CRSwNP remains limited, and there is no research exploring the utility of ERS-related biomarkers in the diagnosis of CRSwNP.

In this study, we integrated 2 GEO datasets, GSE136825 and GSE179265, and conducted immune infiltration analyses. The results revealed that CRSwNP tissues exhibit significantly higher levels of infiltrating resting memory CD4+ T-cells, M2 macrophages, activated dendritic cells, resting mast cells, and neutrophils, while plasma cells, CD8 T-cells, and monocytes were found at lower abundance. This abnormal immune profile suggests a type 2 immune response, which is a prominent feature of CRSwNP^[4]^ and is associated with the presence of nasal polyps. The high infiltration of M2 macrophages and activated dendritic cells may regulate angiogenesis and promote extracellular matrix deposition, potentially leading to tissue remodeling.^[21,32]^ Additionally, research indicates that elevated mast cell infiltration can contribute to eosinophilic inflammation in CRSwNP.^[33]^ Understanding the roles of these immune cells in CRSwNP may guide the development of new diagnostic and therapeutic strategies, paving the way for personalized treatment approaches based on immune profiles. Furthermore, GSVA of the expression profiles between the CRSwNP and control groups revealed significant alterations in pathways related to cellular respiration, oxidative stress-induced apoptosis, and the p53 signaling pathway in the CRSwNP group. Studies have shown that oxidative stress may play a critical role in promoting inflammation and tissue remodeling, which are pathological features of CRSwNP.^[34]^ This aligns with the upregulation of oxidative stress-related pathways observed in our study.

Through differential expression analysis and WGCNA, this study identified sets of DEGs associated with CRSwNP, among which 28 were related to ERS. Machine learning models demonstrated more robust performance with lower error rates in predicting incidence.^[35,36]^ To further identify hub ERS-DEGs, we employed 4 machine learning algorithms (SVM, RF, XGB, and LASSO) and ultimately screened out 3 hub ERS-DEGs. These 3 hub ERS-DEGs showed negative correlations with macrophages M2, dendritic cells activated, mast cells resting, and neutrophils, while exhibiting a positive correlation with plasma cells, which aligns with the results of the aforementioned immune infiltration analysis. Therefore, we hypothesize that the identified 3 hub ERS-DEGs play a significant role in the immune regulation of CRSwNP, contributing to immune imbalance and aberrant inflammatory responses in the disease. They may serve as potential biomarkers closely associated with ERS in CRSwNP and represent promising diagnostic or therapeutic targets.

Finally, we constructed a nomogram clinical prediction model using these 3 hub ERS-DEGs and validated it using DCA, calibration curves, and ROC curves. The results demonstrated that this nomogram model has high accuracy in predicting the risk of CRSwNP occurrence. Additionally, we validated the predictive capability of the model using an external dataset. Nevertheless, it is important to acknowledge that although bioinformatics analysis indicates favorable predictive performance of our clinical prediction model, further confirmation with clinical cases is warranted.

The enzyme encoded by the ATP2A3 gene regulates calcium homeostasis by hydrolyzing ATP. As a cation-transporting ATPase, it functions as a calcium transporter involved in various physiological functions and signaling pathways, playing a critical role in the progression of endometriosis.^[37]^ Furthermore, ATP2A3 has been reported in multiple cancers and is associated with susceptibility to various human malignancies through its regulation of gene transcription and cell proliferation.^[38]^ ACTC1 is a member of the actin family, participating in cell motility, muscle regeneration, and ferroptosis.^[39]^ Experimental studies have confirmed that ACTC1 expression is reduced in CRS, with even lower expression in type 2 CRS compared to non-type 2 CRS, suggesting its potential as a predictive biomarker for type 2 CRS.^[40]^ DES is a muscle-specific intermediate filament protein, mutations of which lead to skeletal myopathies often associated with cardiomyopathy.^[41]^ Additionally, studies have shown decreased expression of DES in the blood of patients with carotid artery stenosis.^[42]^ However, no research has yet explored the role of DES in CRSwNP.

However, it must be pointed out that this study has some limitations. Although we used bioinformatics methods to evaluate 3 hub genes, which may be new targets for ERS-related diagnosis and treatment in CRSwNP, we lack experimental verification results, and the specific role of these hub genes in the pathogenesis of CRSwNP needs further study. Similarly, due to the limited sample size of the external validation datasets, the accuracy of the prediction model based on the hub genes should also be evaluated in patients, which will be explored in future studies. Additionally, due to the limited availability of datasets that encompass various endotypes of CRSwNP, our study did not include specific endotype classifications. Furthermore, although the inferior turbinate and uncinate tissues are anatomically continuous and have been combined in previous studies, the absence of a stratified analysis by tissue source represents another limitation of this study. Therefore, further investigations with larger sample sizes are warranted to validate our findings.

## 5. Conclusion

In conclusion, by integrating multiple bioinformatics approaches including WGCNA, differential expression analysis, and machine learning, this study has consistently identified a group of hub ERS-DEGs closely associated with ERS in CRSwNP. This discovery, converged through multiple computational methods, sheds new light on the potential central role of ERS in the pathogenesis of CRSwNP, providing novel bioinformatics insights and potential therapeutic targets. Additionally, the diagnostic prediction model constructed based on these hub ERS-DEGs preliminarily demonstrates their feasibility as a screening tool. Future research should focus on experimentally validating the functions of these hub genes and exploring their underlying mechanisms, thereby contributing to the optimization of diagnostic and therapeutic strategies for CRSwNP.

## Acknowledgments

The authors express their sincere appreciation to all researchers for sharing and publishing the data.

## Author contributions

**Conceptualization:** Yue Zhai.

**Formal analysis:** Yue Zhai.

**Methodology:** Yue Zhai.

**Writing – original draft:** Yue Zhai.

**Writing – review & editing:** Yue Zhai, Hanli Lu, Qiu Ding.

**Data curation:** Hanli Lu, Qiu Ding.
